# Cryoballoon Ablation of the Pulmonary Veins, the Posterior Wall, the Left Atrial Appendage, and the Mitral Isthmus Guided by Intracardiac Echocardiography–integrated and Three-dimensional Mapping

**DOI:** 10.19102/icrm.2022.130105

**Published:** 2022-01-15

**Authors:** Arash Aryana, Paula Lafortune, Luigi Di Biase, Andrea Natale

**Affiliations:** ^1^Mercy General Hospital and Dignity Health Heart and Vascular Institute, Sacramento, CA, USA; ^2^Albert Einstein College of Medicine and Montefiore Medical Center, Bronx, NY, USA; ^3^Texas Cardiac Arrhythmia Institute, St. David’s Medical Center, Austin, TX, USA

**Keywords:** Cryoablation, cryoballoon, left atrial appendage, mitral isthmus, posterior wall

## Abstract

Here, we describe the first reported case of pulmonary vein, posterior wall, and left atrial appendage (LAA) isolation with concomitant ablation of a mitral isthmus flutter using the cryoballoon, guided by intracardiac echocardiography (ICE)-integrated 3-dimensional mapping in a patient with long-standing persistent atrial fibrillation and intolerance to long-term oral anticoagulation, followed by ICE-guided LAA closure using an endocardial occlusion device. This report illustrates the safety and feasibility of this combined approach. Additionally, it advocates for empiric LAA isolation prior to LAA occlusion, as LAA ablation/isolation can prove challenging in those with existing endocardial LAA occlusion devices should they require subsequent ablations to target arrhythmias or triggers arising from this structure.

## Introduction

Although pulmonary vein (PV) isolation (PVI) remains the cornerstone of atrial fibrillation (AF) ablation,^1^ the success rate associated with cryoballoon PVI alone in patients with persistent AF remains low.^2^ Meanwhile, several studies have demonstrated incremental benefits associated with left atrial posterior wall isolation (PWI),^3–5^ specifically within the region lying between the PVs or the so-called PV component,^6^ as well as electrical isolation of the left atrial appendage (LAA)^7^ in addition to PVI. In this article, we describe the first reported case of concurrent PVI, PWI, and LAA isolation along with mitral isthmus ablation for the treatment of a peri-mitral flutter performed using the cryoballoon guided by intracardiac echocardiography (ICE)-integrated 3-dimensional (3D) mapping, followed by ICE-guided LAA closure using an endocardial occlusion device.

This case illustrates the safety and feasibility of this combined approach. In addition, it advocates for empiric LAA isolation prior to LAA occlusion therapy, as LAA ablation/isolation can prove challenging in a patient with an existing endocardial LAA occlusion device should that patient require subsequent ablations to target LAA-related AF triggers or atrial arrhythmias.

## Case presentation

A 74-year-old woman with a past medical history significant for symptomatic, long-standing persistent AF, refractory to anti-arrhythmic drug therapy and cardioversion, as well as intolerance to long-term oral anticoagulation due to recurrent gastrointestinal bleeding from arteriovenous malformations (CHA_2_DS_2_-VASc score, 4 points; HAS-BLED score, 4 points), in the setting of a dilated left atrium, sleep apnea, systemic and pulmonary hypertension, diabetes mellitus, and chronic lymphocytic leukemia underwent catheter ablation using a cryoballoon and LAA closure using an endocardial LAA occlusion device. The procedure was performed under general anesthesia. Bilateral femoral venous access was obtained under ultrasound guidance. A decapolar catheter (Dynamic XT™; Boston Scientific, Natick, MA, USA) was inserted inside the coronary sinus for left atrial recording and pacing and a duodecapolar catheter (Livewire™; Abbott, Chicago, IL, USA) was placed in the high right atrium/superior vena cava for right atrial recording/pacing and phrenic nerve stimulation. After intravenous systemic anticoagulation, a transseptal puncture was performed and the left atrium and the PVs were mapped by fast anatomical mapping (Carto; Biosense Webster, Diamond Bar, CA, USA). All four PVs were targeted and successfully isolated using a 28-mm cryoballoon (Arctic Front Advance PRO; Medtronic, Minneapolis, MN, USA) followed by PWI, which was guided by ICE. As previously described,^3,5^ ICE image integration (CartoSound; Biosense Webster) allows for direct visualization of the balloon within the 3D map. One or 2 120- to 180-second cryoballoon applications (6 in total) were delivered to each PV using a pre-specified dosing algorithm, guided by time to PVI as validated in a previous study.^8^ During ablation of the right PVs, high-output right phrenic nerve stimulation (≥10 mA) was performed from the superior vena cava. No diminished/loss of phrenic nerve pacing capture was observed. After the completion of PVI, PWI was subsequently performed also using the cryoballoon within the region of the PV component. Our techniques and the specific catheter maneuvers to achieve PWI using this approach have been previously described.^3^ Briefly, a series of overlapping 120-second cryoapplications (13 in total) were delivered at each posterior wall segment. By anchoring the inner lumen circular mapping catheter (Achieve; Medtronic) inside the PVs, the posterior wall was ablated using a segmental, non-occlusive cryoballoon ablation (NOCA) approach.^9^ Briefly, during the ablation of the superior segments of the posterior wall, the inner lumen catheter was anchored inside the superior PVs, whereas the right inferior PV was used for the ablation of the inferior posterior wall segments. Advancing and retracting the inner lumen catheter distally or proximally within the PV allows the operator to position the cryoballoon along the various segments of the posterior wall. As the maximum cooling zone of the cryoballoon spans along its distal hemisphere, the balloon was aligned in such a way that this surface was in direct contact with the targeted tissue. In addition to tactile feel and fluoroscopy, positioning of the cryoballoon along the various posterior wall segments was guided by ICE image integration, which permits the operator to record the precise balloon locations on the posterior wall. Furthermore, the luminal esophageal temperature was monitored throughout the procedure. Based on available data, esophageal temperatures < 15°C were avoided.^10^

**Figure 1:**
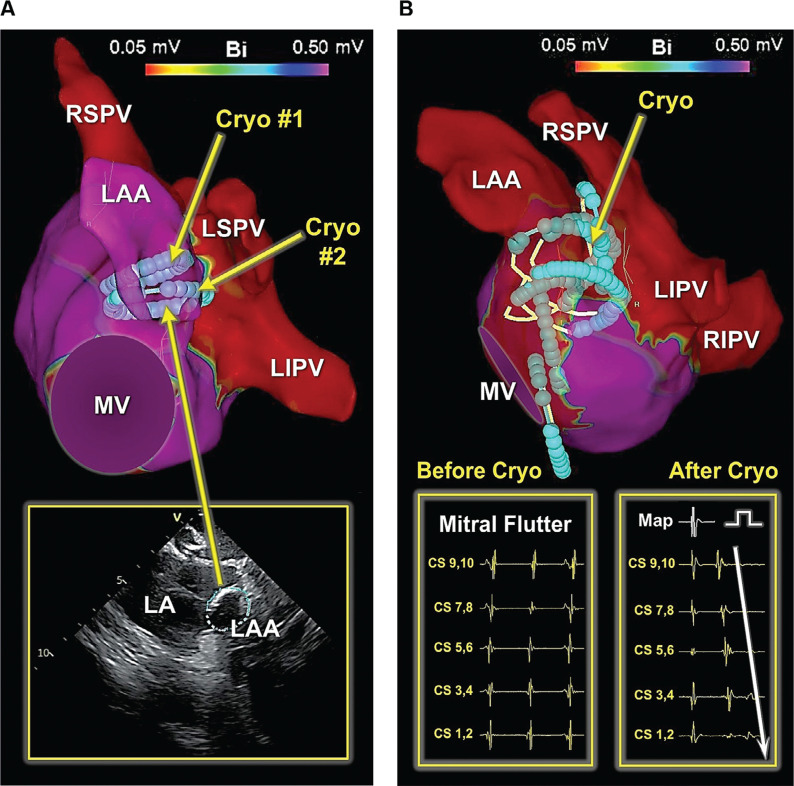
Cryoballoon ablation of the left atrial appendage (LAA) and the mitral isthmus. **A:** A 3-dimensional (3D) bipolar voltage map of the LA depicting the locations of a 180-second cryoapplication (Cryo #1) followed by a subsequent 120-second bonus application (Cryo #2) to achieve LAA isolation. The position of the second cryoapplication is shown on intracardiac echocardiography (ICE) **(inset)**, which is integrated into the 3D map (CartoSound; Biosense Webster), thereby allowing direct visualization of the balloon at the ostium of the LAA. **B:** A 3D bipolar voltage map of the left atrium demonstrating the locations of the cryoballoon applications (cryo) for the ablation of the mitral isthmus. Once again, the balloon locations are directly recorded within the 3D map using ICE image integration. The maximum cooling zone of the cryoballoon spans over its entire distal hemisphere, shown in a turquoise color together with the proximal hemisphere appearing in light yellow. Shown here also are the intracardiac recordings during the mitral isthmus flutter (cycle length, 240 ms) prior to cryoablation **(left lower inset)** and assessment of block across the mitral isthmus post-cryoablation **(right lower inset)** when pacing anterior to the line of block using a diagnostic mapping catheter. The voltage cutoff is set to 0.05 mV in both maps. Abbreviations: CS, coronary sinus; LA, left atrium; LAA, left atrial appendage; LIPV, left inferior pulmonary vein; LSPV, left superior pulmonary vein; MV, mitral valve; RIPV, right inferior pulmonary vein; RSPV, right superior pulmonary vein.

**Figure 2:**
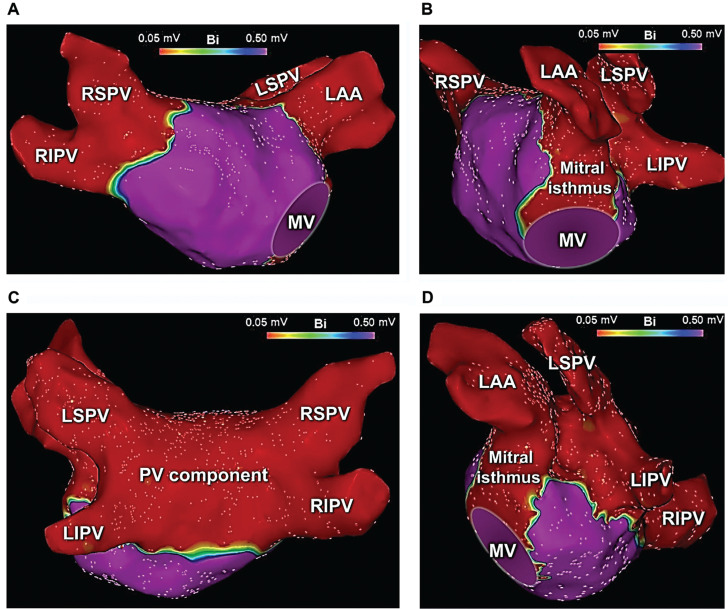
Post-ablation 3-dimensional (3D) voltage maps of the left atrium. **A:** A 3D bipolar voltage map of the left atrium in a right anterior–lateral projection depicting wide-area, antral pulmonary vein (PV) and left atrial appendage ablation/isolation achieved using segmental non-occlusive cryoballoon ablation (NOCA). **B:** A 3D voltage map of the left atrium in a left lateral view created after segmental NOCA of the mitral isthmus using a series of 120-second cryoballoon applications. **C:** A 3D voltage map of the left atrium in a posterior projection illustrating bilateral PV and posterior wall ablation within the region of the PV component. **D:** A 3D voltage map of the left atrium in a left posterolateral projection demonstrating wide-area, antral PV ablation, including the left atrial ridge, the LAA, and the mitral isthmus. The voltage cutoff is set to 0.05 mV in all four maps. Abbreviations: LAA, left atrial appendage; LIPV, left inferior pulmonary vein; LSPV, left superior pulmonary vein; MV, mitral valve; PV, pulmonary vein; RIPV, right inferior pulmonary vein; RSPV, right superior pulmonary vein.

**Figure 3:**
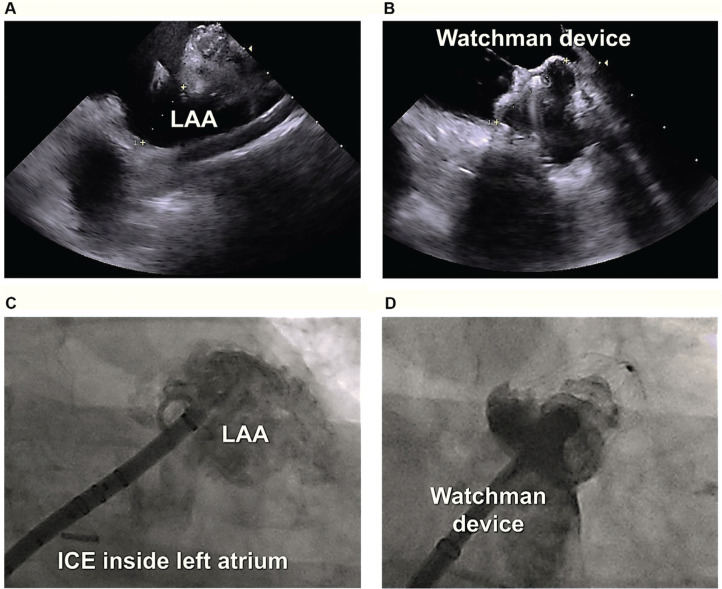
Left atrial appendage (LAA) occlusion using a Watchman™ device guided by ICE. Shown are the LAA size measurements and morphology as demonstrated by ICE from inside the left atrium **(A)** and angiography **(B)**. Endocardial LAA occlusion was subsequently performed using a 27-mm Watchman FLX™ device guided by ICE imaging **(C)** and contrast angiography **(D)**. Abbreviations: ICE, intracardiac echocardiography; LAA, left atrial appendage.

Meanwhile, during cryoballoon ablation of the posterior wall in this patient, the rhythm converted from AF into a mitral isthmus-dependent left atrial flutter with a mean cycle length of 240 ms. As a result, catheter ablation of the mitral isthmus along with complete electrical isolation of the LAA was performed using the cryoballoon. Given the planned LAA occlusion coupled with the existing data in support of a benefit toward improved freedom from recurrent atrial arrhythmias,^7^ we felt that empiric LAA isolation would be advantageous prior to mechanical LAA occlusion as well as eliminating any future potential sources of LAA-related arrhythmias. As such, the LAA was isolated using a single 180-second cryoapplication (time to isolation, 58 seconds). A second, 120-second bonus application was also applied **(Figure 1A)**. High-output left phrenic nerve stimulation (≥10 mA) was performed throughout the ablation using the inner lumen circular mapping catheter from within the LAA. No diminished/loss of phrenic nerve pacing capture was observed during or following ablation. After PVI, PWI, and LAA isolation, the mitral isthmus was similarly targeted using a series of segmental, 120-second cryoapplications. During ablation of the mitral isthmus, the inner lumen circular mapping catheter was placed across the mitral valve, unanchored within the left ventricle. Care was taken not to occlude the valvular orifice. Balloon stability during applications was attained as a consequence of cryo-adhesion. Altogether, seven cryoballoon applications were required to ablate the mitral isthmus and to achieve block across the isthmus **(Figure 1B)**. Upon completion (total cryoablation time, 61 minutes; total procedure time, 164 minutes), the endpoints of wide-area, antral PVI, PWI, LAA isolation, and mitral isthmus ablation were confirmed through detailed 3D mapping **(Figure 2)**, high-output pacing (>10 mA) from multiple sites within the areas of isolation, as well as differential pacing across the mitral isthmus before and after intravenous drug stimulation. Lastly, the patient underwent LAA occlusion **(Figure 3)** using a 27-mm LAA occlusion device (Watchman FLX™; Boston Scientific), guided by ICE. The patient tolerated the procedures well without any acute or long-term adverse events, including no phrenic nerve, vagal, or esophageal complications. Oral anticoagulation was discontinued after eight weeks. The patient has maintained sinus rhythm off anti-arrhythmic drugs and has been free from adverse events on antiplatelet therapy with low-dose aspirin during long-term follow-up.

## Discussion

Previous studies^11,12^ have shown an incremental benefit associated with PWI performed in conjunction with PVI. The rationale for this approach is supported by both anatomical data, which suggest that the PV component shares its embryologic origins with the primordial PVs,^6^ and cellular and structural remodeling data in patients with persistent AF.^11,13^ Moreover, prior reports also point to the presence of AF rotors and spontaneous triggers within the PV component.^14^ Along these lines, several recent studies^3–5^ investigating the outcomes of cryoballoon PVI + PWI have found this approach to be superior to PVI alone in patients with persistent AF. More recently, the authors have also reported the feasibility of mitral isthmus ablation using the cryoballoon for the treatment of a peri-mitral flutter in such a patient.^15^ As illustrated by this case, in the appropriate patient, PVI + PWI can be successfully achieved using the cryoballoon along with catheter ablation of the mitral isthmus and concomitant isolation of the LAA. Empiric ablation and isolation of the LAA in some studies have been similarly shown to improve the long-term freedom from recurrent atrial arrhythmias in patients with long-standing persistent AF.^7^ But more importantly, if endocardial LAA occlusion is planned, consideration may be given to LAA isolation beforehand. Although a redo AF ablation can be safely and effectively performed in a patient with a pre-existing Watchman™ device, if LAA ablation/isolation becomes necessary during a subsequent procedure, this could prove challenging. In fact, if attempted, in some cases, it may actually result in an increased risk of peri-device leak and atrial arrhythmia recurrences.^16^ Thus, when planning a combined approach of persistent AF ablation and LAA occlusion, consideration may be given to empiric ablation and isolation of the LAA prior to implanting the LAA occlusion device.

## Conclusion

As illustrated by this initial report, wide-area, antral PVI, PWI, LAA isolation, and mitral isthmus ablation can be safely and effectively performed concurrently with the cryoballoon using segmental NOCA. This approach can be greatly facilitated using ICE which can also provide direct 3D mapping visualization of the balloon location at various structures. Moreover, if attempted, LAA isolation should ideally be considered prior to a planned mechanical LAA closure using an endocardial occlusion device.

## References

[1] Haïssaguerre M, Jaïs P, Shah DC (1998). Spontaneous initiation of atrial fibrillation by ectopic beats originating in the pulmonary veins. N Engl J Med.

[2] Brooks AG, Stiles MK, Laborderie J (2010). Outcomes of long-standing persistent atrial fibrillation ablation: a systematic review. Heart Rhythm.

[3] Aryana A, Baker JH, Espinosa Ginic MA (2018). Posterior wall isolation using the cryoballoon in conjunction with pulmonary vein ablation is superior to pulmonary vein isolation alone in patients with persistent atrial fibrillation: a multicenter experience. Heart Rhythm.

[4] Nishimura T, Yamauchi Y, Aoyagi H (2019). The clinical impact of the left atrial posterior wall lesion formation by the cryoballoon application for persistent atrial fibrillation: feasibility and clinical implications. J Cardiovasc Electrophysiol.

[5] Aryana A, Allen SL, Pujara DK (2021). Concomitant pulmonary vein and posterior wall isolation using cryoballoon with adjunct radiofrequency in persistent atrial fibrillation. JACC Clin Electrophysiol.

[6] Elbatran AI, Anderson RH, Mori S, Saba MM (2019). The rationale for isolation of the left atrial pulmonary venous component to control atrial fibrillation: a review article. Heart Rhythm.

[7] Di Biase L, Burkhardt JD, Mohanty P (2016). Left atrial appendage isolation in patients with longstanding persistent AF undergoing catheter ablation: BELIEF trial. J Am Coll Cardiol.

[8] Aryana A, Kenigsberg DN, Kowalski M (2017). Verification of a novel atrial fibrillation cryoablation dosing algorithm guided by time-to-pulmonary vein isolation: results from the Cryo-DOSING Study (Cryoballoon-ablation DOSING Based on the Assessment of Time-to-Effect and Pulmonary Vein Isolation Guidance). Heart Rhythm.

[9] Aryana A, Su W, Kuniss M (2021). Segmental nonocclusive cryoballoon ablation of pulmonary veins and extrapulmonary vein structures: best practices III. Heart Rhythm.

[10] Osório TG, Iacopino S, Coutiño HE (2019). Evaluation of the luminal esophageal temperature behavior during left atrium posterior wall ablation by means of second-generation cryoballoon. J Interv Card Electrophysiol.

[11] Segerson NM, Daccarett M, Badger TJ (2010). Magnetic resonance imaging-confirmed ablative debulking of the left atrial posterior wall and septum for treatment of persistent atrial fibrillation: rationale and initial experience. J Cardiovasc Electrophysiol.

[12] Bai R, Di Biase L, Mohanty P (2016). Proven isolation of the pulmonary vein antrum with or without left atrial posterior wall isolation in patients with persistent atrial fibrillation. Heart Rhythm.

[13] Corradi D, Callegari S, Maestri R (2012). Differential structural remodeling of the left-atrial posterior wall in patients affected by mitral regurgitation with or without persistent atrial fibrillation: a morphological and molecular study. J Cardiovasc Electrophysiol.

[14] Lin WS, Tai CT, Hsieh MH (2003). Catheter ablation of paroxysmal atrial fibrillation initiated by non-pulmonary vein ectopy. Circulation.

[15] Aryana A, Gandhavadi M, Bhaskar R, Di Biase L (2021). Cryoballoon ablation of atypical mitral isthmus-dependent left atrial flutter. J Interv Card Electrophysiol.

[16] Turagam MK, Lavu M, Afzal MR (2017). Catheter ablation for atrial fibrillation in patients with Watchman left atrial appendage occlusion device: results from a multicenter registry. J Cardiovasc Electrophysiol.

